# A Brief Instrument to Assess Both Burnout and Professional Fulfillment in Physicians: Reliability and Validity, Including Correlation with Self-Reported Medical Errors, in a Sample of Resident and Practicing Physicians

**DOI:** 10.1007/s40596-017-0849-3

**Published:** 2017-12-01

**Authors:** Mickey Trockel, Bryan Bohman, Emi Lesure, Maryam S. Hamidi, Dana Welle, Laura Roberts, Tait Shanafelt

**Affiliations:** 1 Stanford University, Stanford, CA USA; 2 The Risk Authority Stanford, Palo Alto, CA USA

## Abstract

**Objective:**

The objective of this study was to evaluate the performance of the Professional Fulfillment Index (PFI), a 16-item instrument to assess physicians’ professional fulfillment and burnout, designed for sensitivity to change attributable to interventions or other factors affecting physician well-being.

**Methods:**

A sample of 250 physicians completed the PFI, a measure of self-reported medical errors, and previously validated measures including the Maslach Burnout Inventory (MBI), a one-item burnout measure, the World Health Organization’s abbreviated quality of life assessment (WHOQOL-BREF), and PROMIS short-form depression, anxiety, and sleep-related impairment scales. Between 2 and 3 weeks later, 227 (91%) repeated the PFI and the sleep-related impairment scale.

**Results:**

Principal components analysis justified PFI subscales for professional fulfillment, work exhaustion, and interpersonal disengagement. Test-retest reliability estimates were 0.82 for professional fulfillment (α = 0.91), 0.80 for work exhaustion (α = 0.86), 0.71 for interpersonal disengagement (α = 0.92), and 0.80 for overall burnout (α = 0.92). PFI burnout measures correlated highly (*r* ≥ 0.50) with their closest related MBI equivalents. Cohen’s *d* effect size differences in self-reported medical errors for high versus low burnout classified using the PFI and the MBI were 0.55 and 0.44, respectively. PFI scales correlated in expected directions with sleep-related impairment, depression, anxiety, and WHOQOL-BREF scores. PFI scales demonstrated sufficient sensitivity to detect expected effects of a two-point (range 8–40) change in sleep-related impairment.

**Conclusions:**

PFI scales have good performance characteristics including sensitivity to change and offer a novel contribution by assessing professional fulfillment in addition to burnout.

There is increasing attention in the medical community to the importance of physician well-being as it impacts not only physicians themselves, but also their team members, healthcare organizations, and patients [1–3]. Physicians have higher rates of burnout and lower satisfaction with work-life integration compared to the general population [4]. These rates have worsened in recent years [5]. Burnout and job dissatisfaction are associated with physicians’ intentions to reduce their work hours or leave their organizations [6–8] and are also associated with reduced quality of care, poor patient outcomes [8, 9], medical errors [10–13], risky prescription practices [14, 15], lower patient adherence to physicians’ recommendations [16], and patient dissatisfaction [17, 18].

Increased recognition of the importance of physician well-being and its personal and professional consequences may afford academic psychiatrists a growing opportunity to serve as mental health experts in the design, implementation, and evaluation of efforts to improve well-being among trainees, academic physicians, and other physicians. In addition, academic psychiatry training programs, and the trainees they serve, may benefit from regular assessment of the well-being of their trainees. Survey tools intended to measure physician well-being must be relevant to physicians, easy to administer, reliable, and sensitive enough to identify physicians at risk for burnout and measure the impact of intervention programs on physicians’ professional fulfillment and burnout. There may be particular value in short, reliable, and valid measures that can be used frequently and longitudinally. Existing measures currently used to assess physician well-being meet some but not all of these criteria.

Optimal tools should capture not only aspects of distress but also dimensions of well-being. However, the preponderance of recent physician well-being research has been focused on burnout. The current standard for measuring burnout is the Maslach Burnout Inventory (MBI) [19]. The MBI assesses (1) emotional exhaustion, the state where “as emotional resources are depleted, workers feel they are no longer able to give of themselves at a psychological level”; (2) depersonalization, the development of “callous or even dehumanized perception of others”; and (3) personal accomplishment, the sense of “competence and successful achievement in one’s work with people” [19]. Although the MBI personal accomplishment subscale captures a component of achievement at work, its authors conceived it and researchers often employ it as a reversed-valence component of burnout. A few studies suggest that among physicians, the MBI’s emotional exhaustion and depersonalization subscales are associated with patient care outcomes [20–22].

Widely cited studies on the national prevalence of physician burnout have used the full MBI or a two-item abbreviated version [4, 5] consisting of two MBI items, each found to correlate highly with their respective full MBI emotional exhaustion and depersonalization subscales [23, 24]. Because the MBI asks the respondent to count the frequency of a feeling as far back as a year and even a lifetime, it may not be optimal for assessing changes due to interventions or other factors across time periods shorter than 1 year. Although useful in estimating the prevalence of burnout and reducing response burden, the abbreviated two-item MBI measure conserves the same response options as the full MBI, which limits its utility in assessment of changes across time periods less than 1 year.

Other studies of physician burnout have used a non-proprietary one-item measure of self-defined burnout instead of MBI items [25, 26]. Although it is easy to administer and imposes the lowest response burden, the one-item self-defined burnout measure is scored as a dichotomous variable indicating the presence or absence of burnout and, therefore, is likely to lack the sensitivity to change achievable with continuous scale measurement. The self-defined burnout item also refers to the present without specifying a reference time period.

The Oldenburg Burnout Inventory (OLBI) has also been employed to measure physician burnout in the USA [27, 28]. Assessing emotional exhaustion and general disengagement from work, the OLBI has a well-validated English version [27] and can be found in its entirety in a published report [29]. The Oldenberg Burnout Inventory instructions do not specify a time period to consider when responding to questions indicating burnout. Absence of a time period anchor may complicate interpretation of variance in scores across time points, which will depend on the time periods that respondents independently formulate as they complete the questionnaire.

With exclusive focus on burnout, the MBI, the OLBI, and the one-item measure of burnout render an incomplete picture of physician well-being at work. To address this deficiency, recent research on physician well-being has supplemented assessment of burnout with assessment of professional satisfaction [5, 30, 31]. Satisfaction is one of many possible rewards that are intrinsic to work itself in the practice of medicine. Others include engagement, happiness, or meaningfulness—including meaningful contribution, feeling worthwhile, and professional self-efficacy. We believe an optimal measure would efficiently capture several or all of these intrinsic components of professional fulfillment. Extrinsic rewards (e.g., financial compensation) may also contribute to professional fulfillment but are less robust motivators of engagement and performance [32–34].

To address these gaps, a team of physicians at a large academic medical center, in collaboration with national physician well-being researchers, has developed an alternative measure of physician well-being, the Professional Fulfillment Index (PFI), to assess both burnout (work exhaustion and interpersonal disengagement) and professional fulfillment in physicians (Appendix A.1). To our knowledge, the PFI is the only instrument that includes items that explicitly specify interpersonal disengagement pertaining to patient care. The PFI assesses burnout and professional fulfillment over the previous 2 weeks, facilitating assessment of recent well-being levels and effects of both short- and longer-term interventions. Here, we evaluate the performance of the PFI by assessing internal consistency and test-retest reliability, sensitivity to detect changes over time, and construct validity. We also identify cut-points in PFI scale scores to provide guidance for dichotomization in reporting of professional fulfillment and burnout.

## Methods

In our evaluation of reliability, sensitivity to change, and construct validity of the PFI measurement tool, we use previously published definitions of these terms and their related components [35, 36]. The construct validity of a survey measure is its ability to measure what it is intended to measure. Reliability of a measure refers to its ability to consistently measure the variable it is intended to measure. Sensitivity to change refers to the ability of an instrument to detect changes over time. Face validity refers to a subjective assessment of whether a survey instrument appears to measure the construct it purports to measure. Content validity of a survey instrument refers to the degree to which the survey items assess the breadth of the variable it is intended to measure. Criterion validity refers to the relationships between scores on a measurement tool and other variables that should correlate with the variable these scores quantify. Evaluation of criterion validity includes assessment of convergent validity, discriminant validity, concurrent validity, and predictive validity. Convergent validity is the convergence or correlation between measures that are intended to assess the same variable. Discriminant validity is the divergence or lack of correlation between measures that are intended to assess distinct variables. Concurrent validity refers to the correlation between scores on a measurement tool and other theoretically associated variables measured at the same time. Predictive validity is not assessed in the current study and refers to the correlation between scores on a measurement tool and other theoretically associated variables measured later. Factor validity refers to the degree to which hypothesized structure of a measurement scale is observed in a data set of responses to survey items [37].

### Recruitment and Study Sample

After approval from the corresponding Institutional Review Board, we recruited 250 physicians at an academic medical center to participate in the study, using fliers, posters, and announcements in newsletters sent to all medical staff members. We intended these study advertisements to reach all house-staff (residents and fellows) physicians (approximately 1100) and all physicians on the medical staff (approximately 2500) at this academic medical center. Written informed consent was obtained from all participants. Participants completed the baseline paper survey during a meal that we provided for them at the hospital in an area reserved during the corresponding block of time for this purpose. A $75 gift certificate for food or online merchandise was offered as an incentive for completion of a follow-up survey 2 to 3 weeks later. All participants who completed the baseline survey received an electronic follow-up survey, 2 weeks later, which consisted of a subset of study measures. We sent up to three e-mail reminders to those who had not yet completed the survey before the end of week 3—when we closed the survey. To protect privacy of participants’ data, all data was stored using unique study identifiers instead of personally identifying information. The key linking ID numbers to individuals was kept separately from the data, in a locked cabinet in a locked office.

### Measures

#### Professional Fulfillment Index (PFI) and Previous Face and Content Validity Feedback

The first author and a subgroup of the academic institutions’ Physician Wellness Committee obtained feedback on face and content validity from the entire committee (*n* > 30) at a large university and from one national expert on physician wellness. Reviewers subjectively agreed that the initial set of items captured the essence of burnout as well as the positive valence professional wellness construct the assessment subcommittee named “professional fulfillment.” The first author and physician wellness subcommittee then pilot tested an initial version of the PFI. They used pilot test data to select and refine items included in the initial short-form PFI scale, which they then used to survey the entire medical staff at the same medical center. Six other academic medical centers have since used these measures to survey their physicians. The initial PFI short-form includes four survey questions to measure professional fulfillment and two dimensions of burnout: work exhaustion (four items) and interpersonal disengagement (four items). After consulting with another expert on burnout in physicians to obtain additional feedback on content validity, we added two additional items to the professional fulfillment scale and two additional items to the interpersonal disengagement burnout scale, which this expert believed would optimize face and content validity. We added these new items to the end of their respective scales for testing in this validation study. Results of validation study analyses —repeated with the original shorter four-item versions of the professional fulfillment and interpersonal disengagement scales—are available upon request.

The professional fulfillment scale assesses the degree of intrinsic positive reward the individual derives from his or her work, including happiness, meaningfulness, contribution, self-worth, satisfaction, and feeling in control when dealing with difficult problems at work. The work exhaustion scale assesses symptoms of exhaustion analogous to the domain assessed by the emotional exhaustion scale of the MBI and other burnout scales. Interpersonal disengagement differs from the depersonalization construct measured by some burnout assessment tools by more specifically assessing empathy and connectedness with others—particularly patients and colleagues. Response options are on a five-point Likert scale (“not at all true” to “completely true” for professional fulfillment items and “not at all” to “extremely” for work exhaustion and interpersonal disengagement items). The three PFI scales together (16-items) typically take less than 3 min to complete (Appendix A.1). Each PFI item is scored from 0 to 4, using the associated five-point Likert scale. Scale scores are then calculated by averaging the items scores of all item within each corresponding scale, such that all scale scores also range from 0 to 4.

#### Self-Reported Medical Errors

The first author and colleagues engaged in healthcare risk reduction developed this questionnaire to measure self-reported errors in diagnosis, medication orders, and tests. Response options are in terms of most recent occurrence on a six-point scale from last week to never (Appendix A.2).

#### Previously Validated Measures

To assess correlation and performance of the PFI relative to other established survey tools, the baseline survey included the following measures: the Maslach Burnout Inventory-Human Services Survey (MBI-HSS) [19] (which includes the nested two-item abbreviated MBI measure [24]); the one-item self-defined burnout measure [25]; Patient-Reported Outcomes Measurement Information System (PROMIS) Sleep-Related Impairment [38], Depression, and Anxiety scales [39]; and the WHO Quality of Life WHOQOL-BREF [40].

The self-defined burnout measure is a one-item burnout question that asks respondents to use their own definition of burnout and select the option best describing their current state. There are five response options, with two indicating no burnout (“I enjoy my work. I have no symptoms of burnout.” and “Occasionally I am under stress, and I don’t always have as much energy as I once did, but I don’t feel burned out.”) and three indicating different intensities of burnout (“I am definitely burning out and have one or more symptoms of burnout, such as physical and emotional exhaustion.” and “The symptoms of burnout that I’m experiencing won’t go away. I think about frustrations at work a lot.” and “I feel completely burned out and often wonder if I can go on. I am at the point where I may need some changes or may need to seek some sort of help.”). When scored, the question is treated as a dichotomous variable so that the former is assigned a value of zero and the latter, one. Researchers employed this question in the Physician Worklife Survey; the Minimizing Error, Maximizing Outcome (MEMO) study; and the Healthy Work Place study [10, 12, 25, 41, 42]. It has been validated against complete and partial versions of the MBI-HSS [26, 43].

The PROMIS provides validated questionnaires to measure several patient-centered outcomes over the past 7 days. We used the PROMIS short form v1.0 for depression (4 questions), anxiety (4 questions), and sleep-related impairment (8 questions). The Depression and Anxiety scale items are answered using a five-point Likert scale indicting frequency, from “Never” to “Always.” The Sleep-Related Impairment scale items ask about sleepiness during the day and perceived effects on performance and are answered using a five-point Likert scale indicating intensity, from “Not at all” to “Very much.” The validity of these three PROMIS measures has been demonstrated [38, 39].

World Health Organization Quality of Life Assessment (WHOQOL-BREF) survey is a 26-item version of the longer WHOQOL-100 survey, a cross-cultural instrument to measure how people feel about aspects of their lives. The WHOQOL-BREF measures 4 domains—physical (7 questions), psychological (6 questions), social (3 questions), and environmental (8 questions)—in addition to 2 questions for overall quality of life. For all of the questions, the reference period is the past 2 weeks and response options are on one of 6 different 5-point Likert scales. The WHOQOL-BREF has been tested in 23 countries and demonstrates good psychometric properties [40, 44].

### Analyses

Descriptive statistics were used to determine distribution properties of new and previously validated scales in the current study sample. Face and content validity involve subjective review processes and were addressed prior to this study (see description of the PFI above).

#### Reliability

Cronbach’s alpha (α) for each PFI scale was calculated to estimate internal consistency reliability. To estimate test-retest reliability, we calculated the correlation between PFI scale scores at baseline and follow-up 2 to 3 weeks later in the subsample of participants expected to have stable scores across this time period.

#### Sensitivity to Change

We have noticed high correlations between sleep-related impairment and PFI measures in previous (program evaluation data) samples. Accordingly, those with stable sleep-related impairment scores were expected to have more stable measures of overall burnout and its subscales (work exhaustion and depersonalization), and professional fulfillment over time. In contrast, those with changes in sleep-related impairment scores were considered more likely to experience changes in overall burnout, work exhaustion, depersonalization, and professional fulfillment. We used ROC analyses to test the sensitivity of the PFI to register expected effects of changes of 2 points or greater in sleep-related impairment scores (scale range 8 to 40) from baseline to follow-up 2 to 3 weeks later [36].

#### Criterion Validity

##### Convergent Validity of PFI Scores with Previously Validated Measures

We used Pearson’s correlation coefficients to assess the degree of convergence between PFI scale scores and scores on their most conceptually proximal MBI subscale counterparts.

##### Convergent and Discriminant Validity of PFI Items and Factor Validity:

Principal component analysis with direct oblimin rotation was used to explore the feasibility of separating PFI items into relevant component scales. We used eigenvalues and percentage of combined item set variance explained by each component, along with face validity of item subset groupings by component to determine how many components to retain. Principle component analysis results showing high factor loadings for a set of scale items on only one component provide evidence that the corresponding items are assessing the same underlying variable (convergent validity) as well as evidence that they are assessing something different from sets of items that load highly on other components (divergently validity). The direct oblimin rotation is an oblique rotation, which is appropriate when underlying variables (components) are expected to correlate. An oblique rotation allows for exploration of evidence that item sets measure distinct underlying variables (i.e., pattern matrix loadings) while also accounting for correlations in the underlying variables (i.e., structure matrix loadings). Principle component analysis results that support theoretically hypothesized groupings of items provide initial feasibility evidence for factor validity, which can be tested with confirmatory factor analysis using a different data set.

##### Concurrent Validity:

Pearson’s *r* correlation coefficients were used to estimate association between PFI scales and previously validated burnout measures, self-reported medical errors, sleep-related impairment, depression, anxiety, and quality of life domains. Correlations between PFI scales and previously validated burnout measures suggest evidence for convergent validity, indicating the feasibility of using the PFI to measure burnout. Correlations between PFI scales and other variables measured at the same time help determine concurrent validity—evidence that PFI scales are associated with other wellness variables.

#### Identification of Cut-Point Scores for Dichotomization of Professional Fulfillment and Burnout

Although both burnout and professional fulfillment are continuous rather than binary variables, we are also aware that many real-world uses of the PFI will call for dichotomization of these measures (i.e., indication of who does and who does not have a specific level of “professional fulfillment” and/or “burnout”). ROC analyses were used to determine optimal cut-points for PFI-measured “professional fulfillment” and “burnout,” using the first question of the WHOQOL-BREF (intended to assess overall quality of life) and previous methods for determining dichotomous burnout, respectively. In order to provide evidence of the utility of these classifications, Cohen’s *d* effect size was also calculated for mean differences in medical error rate and depression symptom severity between physicians with vs physicians without burnout above the identified PFI overall burnout scale cut-point. The statistical significance of these mean group differences was estimated using independent samples *t* tests. Using the ROC determined cutoffs for the professional fulfillment (six-item scale) and burnout (ten-item scale) scales of the PFI, we also cross-tabulated the relationship between burnout and fulfillment to determine the degree to which these PFI dimensions were mutually exclusive.We also compared mean WHOQOL-BREF domain scores across PFI categories (burned out, not burned out but not fulfilled, and not burned out and fulfilled).

## Results

### Sample Demographic Data

Of all 250 participants who completed baseline survey measures, 48.8% identified themselves as women, 50.4% identified themselves as men, and 0.8% elected not to answer the question on gender. The majority (51.2%) were 30 to 39, 33.2% were < 30, 8.8% were 40 to 49, 6.0% were ≥ 50 years of age, and 0.8% elected not to answer the question on age category. The majority (52.4%) identified their race as white or Caucasian; 39.6% identified themselves as Asian; 6.4% identified themselves as part of another racial category (i.e., Indian or South Asian, Black or African, or Latino); and 1.6% elected not to answer the question on race. Specialties reported by participants included the following: medicine (26.4%), radiology (11.6%), anesthesia (10.4%), pediatrics (9.6%), surgery subspecialty (7.2%), pathology (5.6%), radiology subspecialty (5.6%), psychiatry (4.8%), surgery (4.0%), medicine subspecialty (3.6%), neurology (2.8%), obstetrics/gynecology (2.4%), pediatric subspecialty (0.8%), and emergency medicine (0.8%). One participant (0.4%) reported two specialties and ten (4.0%) elected not to report specialty. Of all participants, 185 (74%) were house-staff physicians. Of all 250 study participants, 227 (91%) completed the follow-up survey.

### Survey Scale Descriptive Data

Table 1 presents descriptive statistics for PFI scales and self-reported medical error scale. There is either a floor or a ceiling effect (responses observed at the minimum or maximum of a scale score, respectively) for all scales, which was less than 20% in every case.

**Table 1:** Descriptive statistics: Professional Fulfillment Index and the measure of medical errors

Measure (*n*)	Minimum (% at floor)	Maximum (% ceiling)	Mean (SD)	Skewness (SD)	Kurtosis (SD)
Work exhaustion (250)	0.00 (4.4%)	3.75 (0.0%)	1.41 (0.81)	0.50 (0.15)	0.06 (0.31)
Interpersonal disengagement (249)	0.00 (16.1%)	4.00 (0.4%)	1.03 (0.75)	0.59 (0.15)	0.42 (0.31)
Overall burnout scale (249)	0.00 (3.2%)	3.90 (0.0%)	1.18 (0.70)	0.47 (0.15)	0.13 (0.31)
Professional fulfillment (250)	0.33 (0.0%)	4.00 (4.8%)	2.50 (0.80)	− 0.39 (0.15)	− 0.08 (0.31)
Self-reported medical errors (246)	0.00 (8.1%)	4.75 (0.0%)	1.43 (0.96)	− 0.57 (0.16)	0.01 (0.31)

### Reliability

Of the 227 participants who completed measures at both time points, 100 (44.1%) had stable sleep-related impairment scores (≤ 2-point change; scale range 8 to 40) and thus would be expected to have more stable measures of work exhaustion, depersonalization, overall burnout, and professional fulfillment over time (see “Methods”). Table 2 presents scale Cronbach’s alpha and test-retest reliability estimates—correlations between time-one and time-two scores (2 to 3 weeks later)—in the sample (*n* = 100) of participants with stable (defined as change < 2 points; scale range 8 to 40) sleep-related impairment scores across the two time points. These estimates indicate good internal consistency and test-retest reliability for PFI scales.

**Table 2:** Professional Fulfillment Index (PFI) reliability estimates, sensitivity to change, and convergent validity with Maslach Burnout Inventory (MBI) scales

PFI measure	Cronbach’s α	Test-retest reliability	Sensitivity to detect change in sleep-related impairment: correlation	Sensitivity to detect change in sleep-related impairment: AUC (95% CI)	Correlation with closest MBI equivalent
Work exhaustion	0.86	0.80	0.36	0.67 (0.60–0.74)	0.72 (EE)
Interpersonal disengagement	0.92	0.71	0.32	0.64 (0.57–0.72)	0.59 (DP)
Overall burnout scale	0.92	0.80	0.37	0.68 (0.62–0.76)	0.71 (EE + DP)
Professional fulfillment	0.91	0.82	0.28	0.62 (0.54–0.70)	0.46 (PA)

MBI Scale names: EE = emotional exhaustion, DP = depersonalization, PA = personal accomplishment

All correlations are statistically significant (*p* < 0.05)

### Sensitivity to Detect Expected Change

Table 2 also demonstrates correlations between changes in PROMIS sleep-related impairment scores from baseline to follow-up assessment and changes in PFI scores across the same time period. ROC analyses results also presented in Table 2 suggest adequate sensitivity of all PFI scales to register expected effects of changes of two points or greater in sleep related impairment scale across the same time period—baseline to follow-up between 2 and 3 weeks later.

### Criterion Validity*.*

#### Convergent Validity of PFI Scores with Previously Validated Measures

Table 2 presents correlations between PFI scores at time-one and their closest MBI scale score equivalents. Correlations between the conceptually similar PFI and MBI exhaustion scales are high. The correlations between the conceptually similar PFI interpersonal disengagement and the MBI depersonalization scales are also relatively high (≥ 0.5), although to a lesser degree. The correlation is moderate (≥ 0.3 and < 0.5) between the conceptually related but more substantively different PFI professional fulfillment and MBI personal accomplishment scales.

#### Convergent and Discriminant Validity of PFI Items and Factor Validity

We used factor analysis to justify creation of PFI subscales. Kaiser-Mayer-Olkin measure of sampling adequacy (0.93) and Bartlett’s test of sphericity (*p* < 0.001) indicate suitable data for principal component analysis. Eigenvalues for components 1, 2, and 3 were 8.62, 1.77, and 1.15, respectively. The next highest eigenvalue was 0.67, suggesting retention of a three-component measurement model was reasonable. The three components, professional fulfillment, interpersonal disengagement, and work exhaustion sequentially explained 53.9, 11.0, and 7.2% of combined PFI item set variance. Although these cannot be summed meaningfully in the context of oblique rotation results, considered subjectively these estimates suggest the three component measurement model adequately accounts for the majority of variance in the PFI item set. Perhaps most importantly, conceptual meaningfulness was an important part of our decision to retain three factors. The separation of PFI items by these three components is consistent with face validity of the items within each component-based set and provides a conceptually meaningful measurement schema. Table 3 presents the component loading results of principal components analysis. Pattern matrix results demonstrate adequate convergent and discriminant validity of PFI items to measure three components: (1) professional fulfillment (six items), (2) interpersonal disengagement (six items), and (3) work exhaustion (four items). Scale scores for each component were calculated by taking the average score (range 0 to 4) of each of the items within each scale. There were a few (< 5) respondents who left one or more items blank. We calculated the average scale item score for these respondents if they responded to at least 75% of scale items. The two burnout components—work exhaustion and interpersonal disengagement—are highly correlated (*r* = 0.66). Both burnout components are negatively correlated with professional fulfillment (work exhaustion: *r* = −0.59; interpersonal disengagement: *r* = −0.64).

**Table 3:** Principal component analysis results evidence of convergent and discriminant validity of Professional Fulfillment Index items and factor validity feasibility

	Component		
	Professional fulfillment	Interpersonal disengagement	Work exhaustion
Pattern matrix			
Work exhaustion			
A sense of dread when I think about work I have to do	0.15	− 0.11	0.82
Physically exhausted at work	− 0.16	0.19	0.75
Lacking in enthusiasm at work	0.27	0.09	0.62
Emotionally exhausted at work	0.08	0.04	0.82
Interpersonal disengagement			
Less empathetic with my patients	− 0.01	0.87	0.02
Less empathetic with my colleagues	0.19	0.54	0.26
Less sensitive to others’ feelings/emotions	0.02	0.78	0.13
Less interested in talking with my patients	0.00	0.93	− 0.07
Less connected with my patients	0.02	0.93	− 0.07
Less connected with my colleagues	0.19	0.46	0.28
Professional fulfillment			
I feel happy at work	− 0.68	− 0.04	− 0.26
I feel worthwhile at work	− 0.81	0.05	− 0.12
My work is satisfying to me	− 0.86	− 0.07	− 0.00
I feel in control when dealing with difficult problems at work	− 0.72	0.04	− 0.10
My work is meaningful to me	− 0.86	− 0.11	0.16
I’m contributing professionally (e.g. patient care, teaching, research, and leadership) in the ways I value most	− 0.84	− 0.02	0.06
Structure matrix			
Work exhaustion			
A sense of dread when I think about work I have to do	0.53	0.38	0.84
Physically exhausted at work	0.33	0.49	0.76
Lacking in enthusiasm at work	0.65	0.54	0.81
Emotionally exhausted at work	0.54	0.50	0.89
Interpersonal disengagement			
Less empathetic with my patients	0.43	0.87	0.45
Less empathetic with my colleagues	0.60	0.76	0.63
Less sensitive to others’ feelings/emotions	0.48	0.85	0.53
Less interested in talking with my patients	0.42	0.89	0.40
Less connected with my patients	0.45	0.91	0.42
Less connected with my colleagues	0.57	0.70	0.62
Professional fulfillment			
I feel happy at work	− 0.83	− 0.50	− 0.64
I feel worthwhile at work	− 0.85	− 0.41	− 0.53
My work is satisfying to me	− 0.90	− 0.50	− 0.50
I feel in control when dealing with difficult problems at work	− 0.76	− 0.37	− 0.46
My work is meaningful to me	− 0.84	− 0.46	− 0.36
I’m contributing professionally (e.g. patient care, teaching, research, and leadership) in the ways I value most	− 0.82	− 0.41	− 0.40

#### Concurrent Validity

The MBI depersonalization scale and the PFI interpersonal disengagement scale both had moderate correlations with self-reported medical errors (Table 4). The internal consistency reliability estimate for the self-reported medical error scale is questionable (α = 0.62). The relatively low internal consistency in this short four-item scale may be attributable to the small number of scale items, since Cronbach’s alpha depends—in part—on number of scale items. Therefore, we also calculated the mean inter-item correlation for this scale (0.28), which is acceptable [45].

**Table 4:** Concurrent validity: Pearson’s correlations of MBI and Professional Fulfillment Index (PFI) scales with medical errors, sleep-related impairment, depression, anxiety, and World Health Organization Brief Quality of Life (QoL) survey domains

PFI measure	Medical errors	Sleep-related impairment	Depression	Anxiety	Physical QoL	Psychological QoL	Social QoL	Environmental QoL
Work exhaustion	0.15	0.58	0.58	0.57	− 0.55	− 0.60	− 0.32	− 0.42
Interpersonal disengagement	0.33	0.55	0.39	0.42	− 0.42	− 0.44	− 0.28	− 0.37
Overall burnout scale	0.28	0.61	0.53	0.53	− 0.52	− 0.55	− 0.32	− 0.43
Professional fulfillment	− 0.09*	− 0.39	− 0.49	0.46	0.43	0.57	0.32	0.40
MBI emotional exhaustion	0.23	0.59	0.56	0.59	− 0.57	− 0.63	− 0.32	− 0.47
MBI depersonalization	0.43	0.41	0.31	0.37	− 0.30	− 0.37	− 0.25	− 0.38
MBI personal accomplishment	− 0.06*	− 0.27	− 0.30	− 0.33	0.33	0.41	0.20	0.38

*MBI* Maslach Burnout Inventory

*p ≥ 0.05. All other correlations are statistically significant (p < 0.05)

**Fig. 1:**
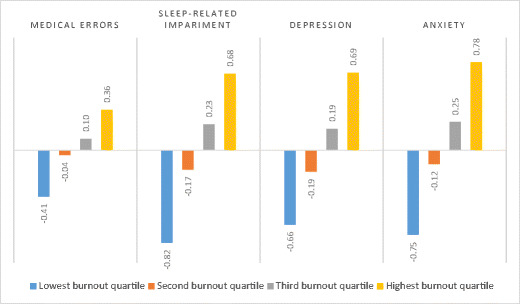
Standardized scores (z-scores) for self-reported medical errors, sleep-related impairment, depression, and anxiety by Professional Fulfillment Scale burnout score quartile

MBI emotional exhaustion, PFI work exhaustion and PFI overall burnout (average score across all PFI burnout items) all had small (> 0.1 < 0.3) but statistically significant correlations with self-reported medical errors. Neither MBI’s personal accomplishment nor PFI professional fulfillment correlated significantly with self-reported medical errors. All MBI and PFI scales correlated moderately or highly in expected directions with PROMIS sleep-related impairment, depression symptom, and anxiety symptom scales, with the exception of the correlation between MBI personal accomplishment and sleep-related impairment, which was − 0.27 (Table 4). Figure 1 demonstrates the dose-response effect on medical errors, sleep-related impairment, depression, and anxiety of PFI burnout scores by quartile. All correlations between MBI and PFI scales with WHOQOL-BREF physical, psychological, social, and environmental domain quality of life scores were moderate to high, with the exception of smaller but statistically significant correlations between social quality of life scores and PFI interpersonal disengagement, MBI depersonalization, and MBI personal accomplishment scores (Table 4).

### ROC Analyses and Cut-Points for PFI Professional Fulfillment and Burnout Scales

To our knowledge, there is no other current validated measure of professional fulfillment. We conducted an ROC analysis using the first item of the WHOQOL-BREF, which is “How would you rate your quality of life?” Response options for this question are “very poor,” “poor,” “neither poor nor good,” “good,” or “very good.” With a response to this item of “very good” set at the positive state, ROC analysis demonstrated the PFI professional fulfillment scale estimated area under the curve (AUC) was 0.81 (95% CI = 0.74–0.78). Professional fulfillment scale sensitivity and specificity for identifying physicians who indicate their quality of life is “very good” using an average-item score cut-point of 3.0 (scale range = 0 to 4) or greater was 0.73 and 0.79, respectively. Note that in this context, sensitivity refers to the portion of participants who test positive for “Quality of Life” who are identified as having “professional fulfillment.” This is different from the term “sensitivity to change” discussed elsewhere in this manuscript, which refers to the ability of a test to detect changes over time.

We also ran three separate ROC analyses for the PFI burnout composite scale (average of all burnout items, including work exhaustion and interpersonal disengagement items), with the positive state comparison set at (1) MBI indicated high emotional exhaustion or high depersonalization, (2) burnout indicated by the West et al. method using two MBI items, and (3) the burnout indicated via the single-item burnout measure [25]. The AUC estimates for the PFI burnout scale estimated by ROC analyses with these other measures of burnout were 0.85 (95% CI = 0.81–0.90), 0.81 (95% CI = 0.76–0.87), and 0.87 (95% CI = 0.82–0.92), respectively. The PFI burnout scale sensitivity in identifying participants who are also identified as experiencing burnout by each of these three previously published methods was 0.72, 0.72, and 0.85 respectively, using an average item score cutoff point of 1.33 or greater (scale range = 0 to 4). Specificity using the same cut-point was 0.84, 0.77, and 0.76, respectively.

Table 5 demonstrates the portion of participants identified as experiencing significant burnout by the PFI burnout scales and by the three previously published methods. Table 5 also demonstrates average differences—and Cohen’s *d* effect size for each average difference—in self-reported medical errors and depression between participants with and without burnout identified by each of these methods. Independent sample *t* tests indicated that mean group differences were statistically significant with one exception; there was no significant difference in self-reported medical error between those identified as experiencing burnout compared to those identified as not experiencing burnout via the single item self-identified burnout assessment method.

**Table 5:** Dichotomized burnout (high versus low) burnout using various instruments and associated differences in medical errors and depression

Measure		Medical errors: with vs without burnout			Depression: with vs without burnout		
	Burnout	Mean (SD)	Mean (SD)	Cohen’s *d*	Mean (SD)	Mean (SD)	Cohen’s *d*
Professional Fulfillment Index, burnout scale	39%	1.74 (1.05)	1.22 (0.84)	0.55	8.14 (3.27)	5.74 (2.29)	0.81
Maslach Burnout Inventory (MBI)^a^	49%	1.63 (1.04)	1.20 (0.82)	0.44	7.89 (3.29)	5.54 (2.08)	0.79
Abbreviated two-Item MBI	41%	1.70 (1.03)	1.23 (0.85)	0.49	7.89 (3.21)	5.90 (2.50)	0.67
Self-defined single item	32%	1.59 (1.04)	1.35 (0.91)	0.25*	8.82 (3.05)	5.71 (2.34)	1.05

*p ≥ 0.05. All other mean group differences are statistically significant (*p* < 0.05)

^a^MBI burnout defined as high score in either emotional exhaustion or depersonalization

There were no significant differences in the portion of house-staff (residents or fellows) and attending physicians experiencing significant burnout, which was 41 versus 37%, respectively [*χ* ^2^ (*df*, 1) = 0.26; *p* = 0.61] using the PFI burnout scale and 50 versus 48% [*χ* ^2^ (*df*, 1) = 0.08; *p* = 0.77] using the MBI. The portion house-staff and attending physicians experiencing significant professional fulfillment was 34% for both groups.

**Fig. 2:**
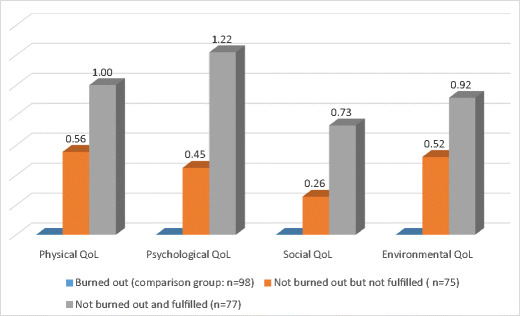
Cohen’s *d* effect-size differences in mean World Health Organization Brief Quality of Life (QoL) domain scores, comparing physicians with and without burnout by professional fulfillment category

Only seven participants (< 3%) were identified by the PFI as experiencing both professional fulfillment and burnout. Of all 250 participants, 98 (39%) were identified by the PFI as experiencing burnout; 75 (30%) were identified as not experiencing burnout but also not experiencing professional fulfillment; and 77 (31%) were identified as experiencing professional fulfillment and not burnout. Figure 2 demonstrates Cohen’s *d* effect size (standard deviation units) differences in average WHOQOL-BREF scores between physicians who were not experiencing burnout but also not experiencing professional fulfillment compared with those who were experiencing burnout, and differences between those experiencing professional fulfillment (and not burnout)—also compared with those who were experiencing burnout.

## Discussion

The Physician Fulfillment Index (PFI) was developed to meet the need for a more robust and balanced approach to assessing wellness variables that (1) are relevant to physicians and are relatively short and easy to use, (2) are well suited to assessment of changes that occur across time in relation to interventions, and (3) include a focus on positive aspects of the role and work of physicians, i.e., professional fulfillment, as well as negative aspects, i.e., burnout. This study involving 250 physicians based at a major academic center demonstrates that the PFI has good internal consistency and test-retest reliability. The PFI correlates well with other widely used measurement tools, while, compared with other tools currently used to assess physicians’ work-related wellness, it is broader and more balanced in its scope. For instance, the PFI work exhaustion scale had a high correlation with the emotional exhaustion scale of the MBI, which is widely used to evaluate burnout variables. Similarly, the PFI interpersonal disengagement also had a high correlation with the related MBI depersonalization scale. Perhaps most importantly, the PFI scales demonstrated sufficient sensitivity to detect the expected burnout and professional fulfillment effects of a two-point or greater (scale range 8 to 40) change in sleep-related impairment, a risk factor for lower physician well-being—particularly among resident physicians [46], who comprised more than two thirds of the current study sample. This psychometric property of the PFI is particularly important because a significant motivator for the development of the PFI was to create a measure sufficiently sensitive to detect recent changes, a measurement property needed when evaluating the effects of an intervention lasting weeks or months rather than years. This finding suggests the PFI is a suitable tool for assessing physician well-being pre- and post-intervention efforts to prevent or ameliorate physician burnout.

Program evaluators and researchers can use PFI scales to assess work exhaustion and interpersonal disengagement separately or can combine these empirically correlated and theoretically related scales to measure overall burnout. This is similar to the Beck depression inventory which includes items that separate in principal components analysis into two highly correlated component scales, which researchers can use to measure cognitive and somatic depressive symptoms separately, but are more often used simply to measure overall depression symptom severity [47]. In contrast, we did not design the PFI professional fulfillment items to assess the opposite end of a burnout spectrum and therefore recommend further psychometric research to elucidate the advantages and disadvantages of combining all three correlated PFI scales into a single index score prior to doing so.

An intriguing finding of this study was the significant correlation of the PFI burnout scales with self-reported medical errors. This finding related to physician behaviors and patient safety is important and warrants further inquiry. Other investigators have found a similar correlation of burnout with self-reported mistakes [10–13]. The PFI now opens the door to research tha explores the potential correlation of physician fulfillment with optimal practices in the care of patients.

### Limitations:

PFI scales, other wellness variables, and medical errors were all assessed via physician self-report in the current study. This introduces potential bias such as recall bias. For example, physicians might be more likely to remember medical errors when experiencing symptoms of burnout. Additional research correlating PFI scores with medical errors or other quality-of-care indicators, not self-reported by physicians, may be helpful.

Although all house-staff (residents and fellows) physicians (approximately 1100) and all physicians on the medical staff (approximately 2500) at this academic medical center were technically invited to participate, we are unable to determine how many saw and read the invitation. We do know that, while the medical staff physicians outnumber house-staff physicians by more than 2 to 1, the sample of participants consisted primarily of house-staff physicians. Typically, house-staff physicians spend the greatest portion of their clinical time in the hospital. It is therefore not surprising that primarily house staff came to the hospital location to participate in the baseline survey during lunch. We recruited a convenience sample at a single site and have no comparative data to determine how those who chose to participate may have differed from those who did not. This sampling method limits our ability to determine the generalizability of study results. On the other hand, the large portion of residents may have added increased sleep related impairment variance across time, facilitating assessment of sensitivity to change in the present study. Nevertheless, additional studies with data across diverse physician samples from multiple sites will provide a more complete understanding of the performance parameters of PFI measures.

In addition, future research would be useful to assess predictive validity of PFI scale scores and to confirm validity of the factor structure implied by the current subscales. A second-order factor structure with a factor for professional fulfillment and a factor for burnout with corresponding subfactors for work exhaustion and interpersonal disengagement is implied by our current use of PFI items. The factor validity of this hypothesized factor structure can be tested via confirmatory factor analysis with an adequately subsequent sample of physician respondents. Studies that track physicians and their patient outcomes longitudinally are also needed, to assess the degree to which PFI scores predict important outcomes such as development of major depressive episodes and career longevity in physicians as well as outcomes in their patients, such as all-cause mortality and hospital readmissions.

Although this initial validation study suggests the PFI measures have good sensitivity to change, we will be even more confident as data from intervention studies using these scales demonstrates this valuable performance parameter directly. Data on change in professional fulfillment will be particularly important, since to our knowledge there are no other published measures of physicians’ professional fulfillment.

### Advancing the Field of Physician Well-Being:

The PFI may be useful for several applications where assessment of physician’s dynamic work-related well-being may be useful. These include assessment of house-staff well-being across different rotations, for example, night float rotations compared rotations with work hours limited to daytime hours, and assessment of well-being following major transitions in training status such as from medical student to intern, intern to resident, and resident to fellow. The PFI may also be useful as a tool for healthcare organizations to assess both burnout and professional fulfillment in their physician staff and to promote efforts to improve well-being and engagement in practicing physicians.

This project represents the first systematic and rigorous effort to develop and explore the psychometric properties of a tool to assess physician fulfillment. Focus on professional fulfillment, a positive valence target for physicians and the healthcare organizations they work in, is an important contribution of the PFI. The PFI is similar to the MBI in that is includes two negative valence scales and one positive valence scale. However, we constructed the professional fulfillment scale to measure several intrinsic fulfillment factors not captured by the MBI Personal Accomplishment Scale. Although the recent focus on physician burnout has fueled much-needed attention to physician well-being, targeting professional fulfillment, as opposed to mere absence of burnout, is an important qualitative addition to comprehensive efforts to improve physician well-being.

On one hand, focus on burnout alone may increase awareness of unnecessary burdens laid on the backs of physicians who stagger under the weight of increasing clerical demands and associated disparate regulatory tasks [48, 49]. On the other hand, public trust in the medical profession may deteriorate in the face of constant depiction of physicians as burned out, no matter whether this is attributed to personal inadequacies, to systematic pathology in medical culture, or both [50]. In any case, widespread burnout among physicians is a real problem warranting systematic intervention.

At this time in the field of medicine, a greater focus on professional fulfillment will strengthen the national discussion of physician health, which has intrinsic importance as well as value to patients across our nation and the quality of our healthcare system. Our study results show that physicians who are experiencing professional fulfillment, in the absence of burnout, have higher scores on quality of life indicators across all domains than other physicians. We hope future research will test our hypothesis that physicians with high professional fulfillment will have clinical care outcomes and clinical quality indicators exceeding those achieved by physicians with either low fulfillment or high burnout or the unfortunate combination of both. We suggest that the PFI may then have even greater utility in that this assessment tool can help measure changes in physician fulfillment and in physician burnout attributable to intervention efforts.

Reliable and valid measures matter a great deal. In designing and testing an instrument for measuring physician fulfillment, we are creating the opportunity for a common approach with greater objectivity and sophistication in endeavors to promote physician well-being, a field that is recognized for its significance in our society but is, as yet, underdeveloped. Tools such as the PFI can help advance this field, for the benefit of physicians, the healthcare teams they lead, and the patients they serve.

## Appendix A.

*Copyright 2016 Board of Trustees of the Leland Stanford Jr. University. All rights reserved. Non-profit organizations are permitted to use this survey instrument without modification for research or program evaluation exclusively. An electronic version of the survey is available by contacting* Wellness.surveyteam@TheRiskAuthority.com*. Any other use of this survey is granted by express written permission of the Stanford WellMd Center by contacting* Wellness.surveyteam@TheRiskAuthority.com

### Appendix A.1 Professional Fulfillment Index

**Table 6:** How true do you feel the following statements are about you at work during the past two weeks?

	Not at all true Score=0	Somewhat true Score=1	Moderately true Score=2	Very True Score=3	Completely true Score=4
a. I feel happy at work	**[ ]**	**[ ]**	**[ ]**	**[ ]**	**[ ]**
b. I feel worthwhile at work	**[ ]**	**[ ]**	**[ ]**	**[ ]**	**[ ]**
c. My work is satisfying to me	**[ ]**	**[ ]**	**[ ]**	**[ ]**	**[ ]**
d. I feel in control when dealing with difficult problems at work	**[ ]**	**[ ]**	**[ ]**	**[ ]**	**[ ]**
e. My work is meaningful to me	**[ ]**	**[ ]**	**[ ]**	**[ ]**	**[ ]**
f. I’m contributing professionally (e.g. patient care, teaching, research, and leadership) in the ways I value most	**[ ]**	**[ ]**	**[ ]**	**[ ]**	**[ ]**

**Table 7:** To what degree have you experienced the following?

**During the past two weeks I have felt…**	**Not at all** Score=0	**Very little** Score=1	**Moderately** Score=2	**A lot** Score=3	**Extremely** Score=4
a. A sense of dread when I think about work I have to do	**[ ]**	**[ ]**	**[ ]**	**[ ]**	**[ ]**
b. Physically exhausted at work	**[ ]**	**[ ]**	**[ ]**	**[ ]**	**[ ]**
c. Lacking in enthusiasm at work	**[ ]**	**[ ]**	**[ ]**	**[ ]**	**[ ]**
d. Emotionally exhausted at work	**[ ]**	**[ ]**	**[ ]**	**[ ]**	**[ ]**
**During the past two weeks my job has contributed to me feeling…**	**Not at all** Score=0	**Very little** Score=1	**Moderately** Score=2	**A lot** Score=3	**Extremely** Score=4
a. Less empathetic with my patients	**[ ]**	**[ ]**	**[ ]**	**[ ]**	**[ ]**
b. Less empathetic with my colleagues	**[ ]**	**[ ]**	**[ ]**	**[ ]**	**[ ]**
c. Less sensitive to others’ feelings/emotions	**[ ]**	**[ ]**	**[ ]**	**[ ]**	**[ ]**
d. Less interested in talking with my patients	**[ ]**	**[ ]**	**[ ]**	**[ ]**	**[ ]**
e. Less connected with my patients	**[ ]**	**[ ]**	**[ ]**	**[ ]**	**[ ]**
f. Less connected with my colleagues	**[ ]**	**[ ]**	**[ ]**	**[ ]**	**[ ]**

### Appendix A.2. Measurement of self-reported medical errors

**Table 8:** Please indicate the most recent time you experienced each of the following things:

	In the last week Score = 5	In the last month Score = 4	In the last 3 months Score = 3	In the last year Score = 2	In my lifetime Score = 1	Never Score = 0
I made a major medical error that could have resulted in patient harm	**[ ]**	**[ ]**	**[ ]**	**[ ]**	**[ ]**	**[ ]**
I made a medical error that did result in patient harm	**[ ]**	**[ ]**	**[ ]**	**[ ]**	**[ ]**	**[ ]**
I ordered the wrong medication	**[ ]**	**[ ]**	**[ ]**	**[ ]**	**[ ]**	**[ ]**
I ordered the wrong lab test	**[ ]**	**[ ]**	**[ ]**	**[ ]**	**[ ]**	**[ ]**
